# Response of MiRNA-22-3p and MiRNA-149-5p to Folate Deficiency and the Differential Regulation of *MTHFR* Expression in Normal and Cancerous Human Hepatocytes

**DOI:** 10.1371/journal.pone.0168049

**Published:** 2017-01-03

**Authors:** Chao Li, Juan Ni, Yao-Xian Liu, Han Wang, Zi-Qing Liang, Xu Wang

**Affiliations:** School of Life Science, Engineering Research Center of Sustainable Development and Utilization of Biomass Energy, Ministry of Education, Yunnan Normal University, Kunming, Yunnan, People’s Republic of China; National Institutes of Health, UNITED STATES

## Abstract

**Background/Aims:**

Folic acid (FA) is a core micronutrient involved in DNA synthesis/methylation, and the metabolism of FA is responsible for genomic stability. MicroRNAs may affect gene expression during folate metabolism when cellular homeostasis is changed. This study aimed to reveal the relationship between FA deficiency and the expression of miR-22-p/miR-149-5p and the targeted regulation of miR-22-3p/miR-149-5p on the key folate metabolic gene *Methylenetetrahydrofolate reductase* (*MTHFR*).

**Methods:**

Normal (HL-7702 cells) and cancerous (QGY-7703 cells) human hepatocytes were intervened in modified RPMI 1640 with FA deficiency for 21 days. The interaction between *MTHFR* and the tested miRNAs was verified by Dual-Luciferase Reporter Assays. The changes in the expression of miR-22-3p/miR-149-5p in response to FA deficiency were detected by Poly (A) Tailing RT-qPCR, and the expression of *MTHFR* at both the transcriptional and translational levels was determined by RT-qPCR and Western blotting, respectively.

**Result:**

MiR-22-3p/miR-149-5p directly targeted the 3’UTR sequence of the *MTHFR* gene. FA deficiency led to an upregulation of miR-22-3p/miR-149-5p expression in QGY-7703/HL-7702 cells, while the transcription of *MTHFR* was decreased in QGY-7703 cells but elevated in HL-7702 cells. Western blotting showed that FA deficiency resulted in a decline of the *MTHFR* protein in QGY-7703 cells, whereas in HL-7702 cells, the *MTHFR* protein level remained constant.

**Conclusion:**

The results suggested that miR-22-3p/miR-149-5p exert different post-transcriptional effects on *MTHFR* under conditions of FA deficiency in normal and cancerous human hepatocytes. The results also implied that miR-22-3p/miR-149-5p might exert anticancer effects in cases of long-term FA deficiency.

## Introduction

MicroRNAs (miRNAs) are small endogenous noncoding RNAs (19–24 nucleotides in length) that have emerged as key negative regulators that target gene expression at the post-transcriptional level [1, 2]. In humans, 2578 mature miRNA sequences are predicted to exist[3]. MicroRNAs have multiple targets, and several miRNAs can also meticulously regulate the same gene simultaneously. MiRNAs contribute to various aspects of animal development and/or human disease[4]. The dysregulation of miRNAs is responsible for one-carbon metabolism (OCM, or folate metabolism), which results in an imbalance of DNA synthesis, methylation, amino acid metabolism and cellular replication. Imbalances in these events are frequently related to an increased risk of various diseases, such as hepatocellular carcinoma (HCC), as some miRNAs act as oncogenes, which leads to the dysregulation of their target gene products in tumors[5, 6].

*Methylenetetrahydrofolate reductase* (*MTHFR*) serves as a key shift gene between DNA methylation/synthesis in OCM through the conversion of 5, 10-methyltetrahydrofolate to 5-methyltetrahydrofolate (5-MTHF). 5-MTHF lends a methyl group to homocysteine to generate methionine, which participates in methylation. In contrast, the loss of a methyl group from 5-MTHF results in the generation of tetrahydrofolate, which participates in thymine synthesis. Abnormal *MTHFR* function is related to low folate status in plasma/red blood cells, which results in the accumulation of plasma homocysteine. Increased plasma homocysteine levels have been indicated to enhance in vivo and in vitro oxidative stress (*P53* oxidative lesions)/endothelial dysfunction. Reduced *MTHFR* activities are associated with a high-risk for the development of HCC and are correlated with lower risks for late-stage HCC and a favorable survival of patients with HCC [7–9].

Hepatocellular carcinoma (HCC) is the fifth most common cancer worldwide and is the third most frequent cause of cancer-related death (GLOBOCAN database, IARC, France, 2012). The incidence of HCC is increasing worldwide[10], and the liver is the major site of metabolism of substances such as chemicals/drugs, alcohol, and environmental pollutants. Thus, the liver is sensitive to deficiencies in micronutrients. One of these micronutrients is folate (or folic acid), which plays an essential role in liver physiology.

Folic acid (FA), which is a water-soluble B vitamin found abundantly in fresh fruits, leafy green vegetables, whole grains, yeast, lima beans, liver, and other organ meats, has been targeted as a potential cancer-preventative agent.[11] Folate functions to provide one-carbon groups for DNA synthesis/methylation and the epigenetic control of gene expression [11–13]. Since the liver is the major site of folate storage and is, therefore, susceptible to folate deficiency, deprivation of this micronutrient may contribute to chromosomal breaks, aneuploidy and deleterious alterations in gene expression within hepatocytes; this, in turn, leads to genetic instability and carcinogenesis [14–17]. For instance, the impact of folate deficiency on biomarkers of telomeres provides evidence of dysfunctional long and short telomeres as a cause of telomere critical length/chromosome instability. [18] A methyl-deficient diet (lack of folate, choline and methionine) results in altered methylation patterns in the *P53* gene in hepatocytes, which have been implicated in an increased risk for hepatocarcinogenesis and/or progression of liver cancer [19–23]. A previous clinical study showed an inverse correlation between the serum folate level and tumor size, multiplicity and metastasis during HCC progression, which was categorized as stage I through IV. Another larger cohort study found that a higher folate level in red blood cells was associated with a reduced risk of hepatocarcinogenesis. The study by Ho CT et al. demonstrated that folate deficiency-triggered redox pathways conferred drug resistance and that folate supplementation may enhance the efficacy of chemotherapy in HCC [24–26]. Moreover, it has been suggested that folate may play critical roles in the prevention of tumorigenesis in different cancer types [27–29], and preliminary studies have indicated a correlation between miR-22/miR-149 and *MTHFR* [30, 31]. These results have implicated the significance of folate status in the carcinogenesis of HCC and other cancers. However, the molecular mechanism that links FA deficiency to *MTHFR*, which leads to the development of liver cancer, remains to be elucidated.

## Materials and Methods

### Cell culture and intervention

The normal human hepatocyte cell line HL-7702 (cat # GNHu 6, Cell Bank of the Chinese Academy of Sciences) and the human hepatocellular carcinoma cell line QGY-7703 (cat # TCHu 43, Cell Bank of the Chinese Academy of Sciences) were cultured in RPMI 1640 medium (GIBCO, NY, USA) supplemented with 8% dialyzed fetal bovine serum (GIBCO, NY, USA) and 1% penicillin/streptomycin (GIBCO, NY, USA). L-Glutamine (1%; GIBCO, NY, USA) was added to the culture medium immediately before use. The cells were plated at a density of 5×10^5^ cells in a 25 cm^2^ culture flask (Costar, Corning, NY, USA). The medium was replaced every 2–3 days for 21 days. All cultures were maintained at 37°C in a humidified atmosphere of 5% CO2.

An in vitro model of chronic FA deficiency was established using the normal human hepatocyte cell line HL-7702 and the hepatocellular carcinoma cell line QGY-7703. The intervening medium was modified from commercial RPMI 1640 medium and folate-free RPMI 1640 medium by changing the FA concentration and using either 0 nM or 22.6 nM FA. The cells were cultured for 21 days in RPMI-1640 medium with modified FA concentrations as follows: FA-free (0 nM), FA-deficient (22.6 nM), or control (2,260 nM). The human HL-7702 and QGY-7703 cell lines were selected because folate deficiency has been identified as an important risk factor for HCC, and alterations in the expression of miRNAs have been shown to be prominent events during the early stages of liver carcinogenesis [32]. Su YH et al. [33] showed that a folate-deficient tumor microenvironment promoted epithelial-to-mesenchymal transition in hepatocytes. Chem CL et al.[34]demonstrated that FA deficiency induced OS and apoptosis in the well-differentiated HCC cell line HepG2. In previous reports, Wu C et al.[31] confirmed the targeting of the 3’UTR of *MTHFR* by miR-149-5p.

### Poly (A) tailing RT-qPCR miR-22-3p and miR-149-5p expression analyses

MiRNAs were purified using a miRNA Isolation Kit (Omega, USA). Polyadenylation and reverse transcription were performed using a miRcute Plus miRNA first-strand cDNA Synthesis Kit (TIANGEN). For poly (A) Tailing RT-qPCR analyses, we used a miRcute miRNA qPCR Detection Kit (SYBR Green). All results were normalized to the expression of U6 small nuclear RNA according to the 2^-ΔΔCt^ cycle threshold method; the error bars indicate the standard deviation. All of the miRNA primers used in our study were purchased from the TIANGEN Human miRNA Specific qPCR Primer Sets (TIANGEN). The PCR reaction was performed in an ABI Step One Plus PCR System (Applied Biosystems, USA).

### Vector construction and dual-luciferase reporter assays

Candidate targets were first determined using target prediction engines such as TargetScan and miRDB. When compared with the targets predicted by TargetScan, miR-22-3p and miR-149-5p target sites were found to be conserved in mammals. These included a predicted target site (miR-22-3p) within the *MTHFR* 3’UTR that is ranked in the 99th percentile according to the TargetScan scoring criteria; however, this seed region was subsequently shown to be a pseudo target site, and thus those results are not shown here. When compared with targets predicted by miRDB, the *MTHFR* 3’UTR also harbored the same five additional poorly conserved miR-22-3p target sites and the same three additional poorly conserved miR-149-5p target sites. This indicates a higher likelihood of miR-22-3p and miR-149-5p targeting functionality. The Dual-Luciferase assay was performed using a Dual-Luciferase Reporter Assay System (psiCHECK-2 vector, Promega). Both 921-bp (miR-22-3p) and 864-bp (miR-149-5p) fragments of the *MTHFR* 3’UTR were amplified from genomic DNA using the following primer sequences: forward, 5’-GGGTTAAGTATGAGGTGAAATGG-3’, reverse, 5’-GACAAACGGTGTCTGAAGTGC-3’ (921 bp); forward, 5’-GGTTGTTGCCAACTAAGCCC-3’, reverse, 5’- TCCAGGGAGTGATGACAGAG-3’ (864 bp). The PCR products were inserted into the psiCHECK-2 vector downstream of the Renilla luciferase gene sequence. All of the constructs were verified by DNA sequencing. The psiCHECK-MUT was constructed through site-directed mutagenesis using a Site-directed Mutagenesis Kit (TransGen, Beijing, China). The primer sequences used for the site-directed mutagenesis were as follows: forward 5’- GTTAAGGTGGGTCCAGGAAGTTGAATGCTCTTAGT -3’, reverse 5’-TTCAACTTCCTGGACCCACCTTAACCTCTTGTGAC -3’ (921bp); forward 5’- CCAAGGCAGCCTCCAGCATTGGCCTGGGACTCC -3’, reverse 5’-CAATGCTGGAGGCTGCCTTGGTTCGAGGGCTTAG -3’ (864 bp). The constructs were verified by sequencing and did not contain any other sequence variations. The HEK293 cell line was cultured in DMEM (GIBCO, NY, USA) with 10% FBS (GIBCO, NY, USA). HEK-293 cells were plated in 24-well culture plates (Costar, Corning, NY, USA) at a density of 1×10^5^ cells per well. Lipofectamine 2000 (Invitrogen, USA) was used to transfect the cells with 0.8 μg per well of psiCHECK-*MTHFR* 3’UTR or psiCHECK-MUT reporter construct, hsa-miR-22-3p/has-miR-149-5p mimic or controls (all at 100 nM, RIBOBIO, Guangzhou, China). Then, 24 hours after transfection, the cells were washed three times in PBS and were lysed with passive lysis buffer. Firefly luciferase and Renilla luciferase activity were determined using a dual-luciferase reporter assay system (Promega, USA) and were measured in a Varioskan LUX plate reader (Thermo).

### Real-time mRNA expression analyses

Total RNA was isolated from cells with a miRNA Isolation Kit (Omega, USA), and its quality was assessed using a Nano Photometer (IMPLEN, Germany). RNA samples were diluted to 1 μg/μL. A reverse transcription reaction was performed (Reverse Transcriptase Kit, Takara, Dalian, China). For quantitative real-time qPCR (RT-qPCR) analyses, we used a SYBR RT-PCR Kit (KAPA, MA, USA). The primers for *MTHFR* were as follows: 5’-CACTACGGTGGGCTGGATGA-3’ (forward) and 5’-GCTCCGGGTTAATTACCACCTTG-3’ (reverse). The primers for Programmed cell death protein 4 (PDCD4) were as follows: 5’-ATGAGCACAACTGATGTGGAAA-3’ (forward) and 5’-ACAGCTCTAGCAATAAACTGGC-3’ (reverse). The primers for Tumor protein p53-inducible nuclear protein 1 (TP53INP1) were as follows: 5’- GCACCCTTCAGTCTTTTCCTGTT-3’ (forward) and 5’- GGAGAAAGCAGGAATCACTTGTAT -3’ (reverse). The primers for GAPDH were as follows: 5’-GGCACAGTCAAGGCTGAGAATG-3’ (forward) and 5’-ATGGTGGTGAAGACGCCAGTA-3’ (reverse). The relative expression level was normalized to that of GAPDH using the ^2-ΔΔCt^ cycle threshold method.

### Immunoblot analyses

The cells were disrupted in cell lysis buffer (cat# P0013, Beyotime, China) supplemented with PMSF (Biosharp, China) and centrifuged for 15 min at 4°C. The protein concentrations were then determined by a BCA protein assay (Beyotime, China). Then, 30 μg of protein were resolved by SDS-PAGE using 10% Tris-glycine gels. Separated proteins were transferred to polyvinylidene fluoride (PVDF) membranes (Millipore, MA, USA) and blocked for 2 h at room temperature with 5% skim milk/TBST (Tris-Buffered Saline with Tween-20). After incubation with an anti-human *MTHFR* (1:2000; Abcam, Cambridge, MA, USA) primary antibody under blocking conditions, proteins were detected using an anti-mouse HRP-conjugated secondary antibody (1:10,000; Vazyme, China) and enhanced chemiluminescence (ECL) (Millipore, MA, USA). The quantification of immunoreactivity was performed by densitometric analysis using Image J. *MTHFR* protein levels were normalized to those of GAPDH (1:5000; Abcam, Cambridge, MA, USA). GAPDH proteins were detected using an anti-rabbit HRP conjugated secondary antibody (1:10,000; Abcam, Cambridge, MA, USA).

### Statistical analysis

The data were analyzed using the Statistical Package for Social Sciences version 21.0 (SPSS, Chicago, IL, USA). Student’s t-test for independent samples was used to compare the levels of *MTHFR* protein, mRNA or miRNA among the samples. All of the statistical hypothesis tests were two-sided, and a P value<0.05 was considered statistically significant.

## Results

### Expression of miR-22-3p and miR-149-5p is upregulated under conditions of FA deficiency

**Fig 1:**
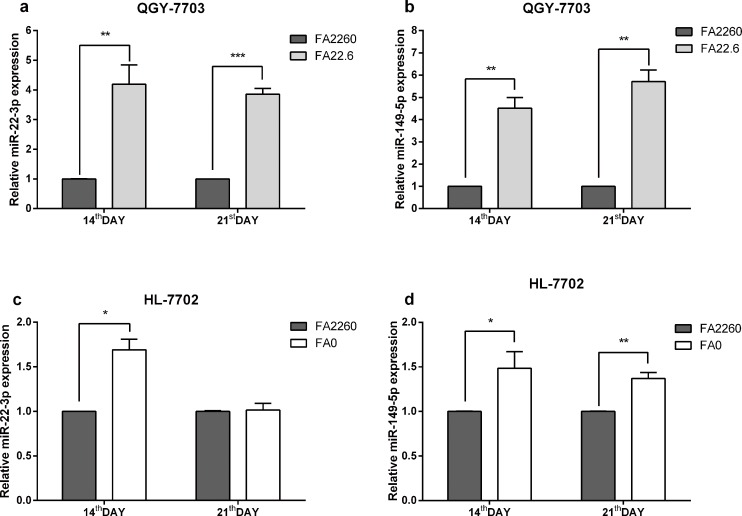
The expression of miR-22-3p and miR-149-5p in QGY-7703 and HL7702 cells on the 14^th^ day and the 21^st^ day under conditions of FA deficiency/culture in the FA-free medium; U6 served as an internal reference. (a) FA deficiency induced an increase in the expression of miR-22-3p in QGY-7703 cells on the 14^th^/21^st^ day. (b) FA deficiency induced an increase in the expression of miR-149-5p in QGY-7703 cells on the 14^th^/21^st^ day. (c) FA-free medium induced an increase in the expression of miR-22-3p in HL-7702 cells on the 14^th^ day. (d) FA-free medium induced an increase in the expression of miR-149-5p in HL-7702 cells on the 14^th^/21^st^ day.

To identify the roles of miR-22-3p and miR-149-5p in the development of HCC under conditions of FA deficiency, we analyzed the expression level of miR-22-3p by Poly(A) Tailing quantitative real-time PCR (RT-qPCR) in the HCC cell line QGY-7703 on the 14^th^ and 21^st^ day after the start of FA deficiency (the cells could not survive in the FA-free RPMI 1640 medium); a matched normal hepatocyte cell line (HL-7702) was also examined. The Poly (A) Tailing RT-qPCR analyses showed that the expression of miR-22-3p under conditions of FA deficiency was increased 4.19-fold (P<0.01, 14^th^ day) and 3.86-fold (P<0.001, 21^st^ day) compared with the control. The results of this assay also showed that the expression of miR-149-5p was increased 4.51-fold (P<0.01, 14^th^ day) and 5.71-fold (P<0.01, 21^st^ day) under conditions of FA deficiency compared with the control. We also determined the expression of miR-22-3p in the normal hepatocyte cell line HL-7702. It was shown that miR-22-3p and miR-149-5p were also upregulated 69% (P<0.05, 14^th^ day) and 48% (P<0.05, 14^th^ day), respectively, miR-149-5p were upregulated 37% (P<0.01, 21^st^ day) when HL-7702 cells were grown in the FA-free medium compared with the control. Notably, the function of miR-22-3p in HL-7702 cells might be replaced by the increase in miR-149-5p on the 21^st^ day, and we also found a potential substitution effect between miR-22-3p and miR-149-3p (Fig 1) Our results indicated that FA deficiency induces miR-22-3p and miR-149-5p upregulation and suggested the inhibition of *MTHFR* expression by miR-22-3p and miR-149-5p in QGY-7703 and HL-7703 cells.

### The 3’UTR of *MTHFR* is directly targeted by miR-22-3p and miR-149-5p

**Fig 2:**
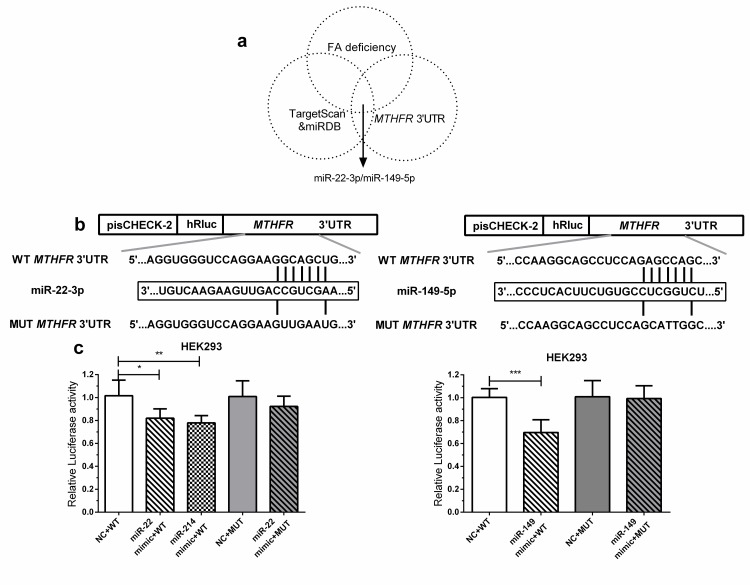
Prediction and validation of the 3’UTR of the *MTHFR* gene as a target of miR-22-3p. (a) In vertebrates, the *MTHFR* gene contains miR-22-3p and miR-149-5p seed regions, which were predicted by miRNA target prediction tools (TargetScan and miRDB). (b) The sequence of miR-22-3p/miR-149-5p (middle) corresponded to the 3’UTR of *MTHFR* (top) but did not correspond completely when the 3’UTR of *MTHFR* was mutated (bottom). (c) MiR-22-3p/miR-149-5p inhibited the dual-luciferase reporter activity of the wild-type but not the mutated 3’UTR of *MTHFR*. HEK293 cells were co-transfected with a dual-luciferase reporter vector (pisCHECK-2) wild-type or mutant *MTHFR* 3’UTR and with an miR-22-3p mimic, an miR-214-3p mimic, an miR-149-5p mimic or miR-NC, as indicated. After 24 h, Renilla luciferase activity was measured and normalized to the firefly luciferase activity. The data are significant at P<0.05.

To determine whether *MTHFR* is a direct target of miR-22-3p and miR-149-5p, wild-type and mutant sequences of the 3’UTR of *MTHFR* were cloned downstream of the Renilla luciferase coding region in a psiCHECK-2 vector. HEK293 cells were then co-transfected with these constructs along with psiCHECK-*MTHFR* 3’UTR (miR-22-3p/miR-149-5p), psiCHECK-MUT (miR-22-3p/miR-149-5p), miR-22-3p mimic, miR-214-3p mimic (same 3’UTR as miR-22-3p, as a positive control[35]), miR-149-5p mimic or miR-NC. The relative luciferase activity was reduced by 18% (P = 0.014), 22% (P = 0.003), and 30% (P<0.001) in psiCHECK-2 vectors with wild-type *MTHFR* 3’UTRs but not in those with the mutant 3’UTR (P>0.05) (Fig 2) The observation that miR-149-5p targeted this seed region was consistent with the results of Wu C et al.[31]. Here, the direct binding of miR-22-3p to the 3’UTR of *MTHFR* has been demonstrated for the first time. In summary, the negative regulation of *MTHFR* by miR-22-3p and miR-149-5p is relevant in this context.

### Expression of *MTHFR* is affected under conditions of FA deficiency

**Fig 3:**
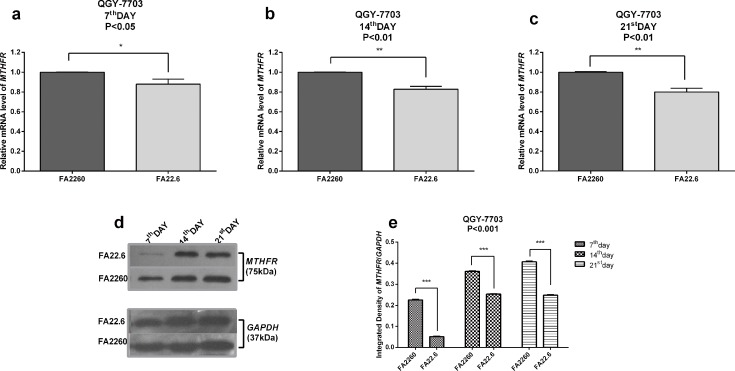
mRNA expression and protein level of *MTHFR* in QGY-7703 cells under conditions of FA deficiency (7^th^, 14^th^ and 21^st^ day); GAPDH served as an internal reference. (a) RT-qPCR assays revealed the mRNA expression of *MTHFR* in QGY-7703 cells under conditions of FA deficiency on the 7^th^ day. (b) RT-qPCR assays revealed the mRNA expression of *MTHFR* in QGY-7703 cells under conditions of FA deficiency on the 14^th^ day. (c) RT-qPCR assays reveal the mRNA expression of *MTHFR* in QGY-7703 cells under conditions of FA deficiency on the 21^st^ day. (d, e) Western blot assays showed the relative protein expression of *MTHFR* in QGY-7703 cells under conditions of FA deficiency (7^th^, 14^th^, 21^st^ day); GAPDH served as an internal reference.

**Fig 4:**
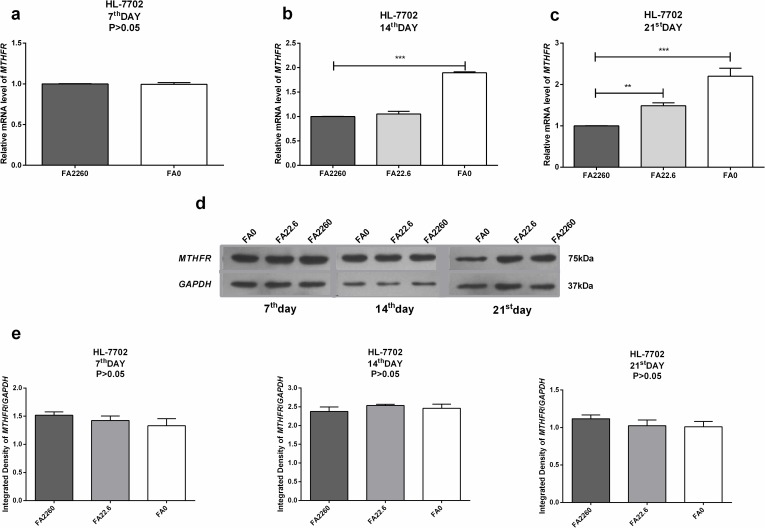
The mRNA expression and protein level of *MTHFR* in HL-7702 cells under conditions of FA deficiency (7^th^, 14^th^ and 21^st^ day); GAPDH served as an internal reference. (a) RT-qPCR assays revealed the mRNA expression of *MTHFR* in HL-7702 cells under conditions of FA deficiency on the 7^th^ day. (b) RT-qPCR assays revealed the mRNA expression of *MTHFR* in HL-7702 cells under conditions of FA deficiency/FA-free medium on the 14^th^ day. (c) RT-qPCR assays revealed the mRNA expression of *MTHFR* in HL-7702 cells under conditions of FA deficiency/FA-free medium on the 21^st^ day. (d, e) Western blot assays showed the relative protein expression of *MTHFR* in HL-7702 cells under conditions of FA deficiency/FA-free medium (7th, 14^th^, 21^st^ day); GAPDH served as an internal reference.

To explore the miR-22-3p and miR-149-5p induced difference in expression of *MTHFR* between HCC cells and normal hepatocytes, the relative expression of *MTHFR* in hepatocellular carcinoma cells under conditions of FA deficiency was markedly lower in 12% (P<0.05, 7^th^ day), 17% (P<0.01, 14^th^ day) 20% (P<0.01, 21^st^ day) of cells compared with the control. Moreover, the Western blotting result showed that the expression of MTFHR was remarkably lower in cells cultured under conditions of FA deficiency compared with the control (P<0.001, 7^th^, 14^th^, 21^st^ day). Notably, the western blotting result showed that FA deficiency after 7 days led to a significant reduction in the levels of *MTHFR* compared with the other time points; we supposed that this observation might be associated with sensitivity to FA deficiency. (Fig 3) The relative expression was not changed between normal hepatocytes cultured in the FA-free medium and those cultured in control medium by the 7^th^ day. However, the relative expression levels of *MTHFR* mRNA were significantly increased 1.89-fold (P<0.001) on the 14^th^ day after culture in the FA-free medium and were clearly increased by 1.48-fold (P<0.01, FA deficiency) and 2.20-fold (P<0.001, FA free), respectively, on the 21st day. On the contrary, western blotting revealed no marked changes between cells cultured under conditions of FA deficiency/FA-free medium and the control. (P>0.05) (Fig 4) Our study indicated that the increased expression of miR-22-3p (3.86–4.19-fold) and miR-149-5p (4.51–5.71-fold) might cause a decrease in *MTHFR* mRNA expression in HCC cells. Our study also indicated that the elevated expression of miR-22-3p (69%) and miR-149-5p (37–48%) might maintain the translational level of *MTHFR* in normal hepatocytes by directly targeting and inhibiting the overexpression of *MTHFR* mRNA in conditions of FA deficiency. *MTHFR* remained constant in normal Hepatocytes cells, and thus, it maintained cellular homeostasis of *MTHFR* and resisted to the FA deficiency. In contrast, the declined expression of *MTHFR* caused HCC cells more vulnerable to the FA deficiency.

### Expression of *PDCD4/TP53INP1* is affected under conditions of long term FA deficiency

**Fig 5:**
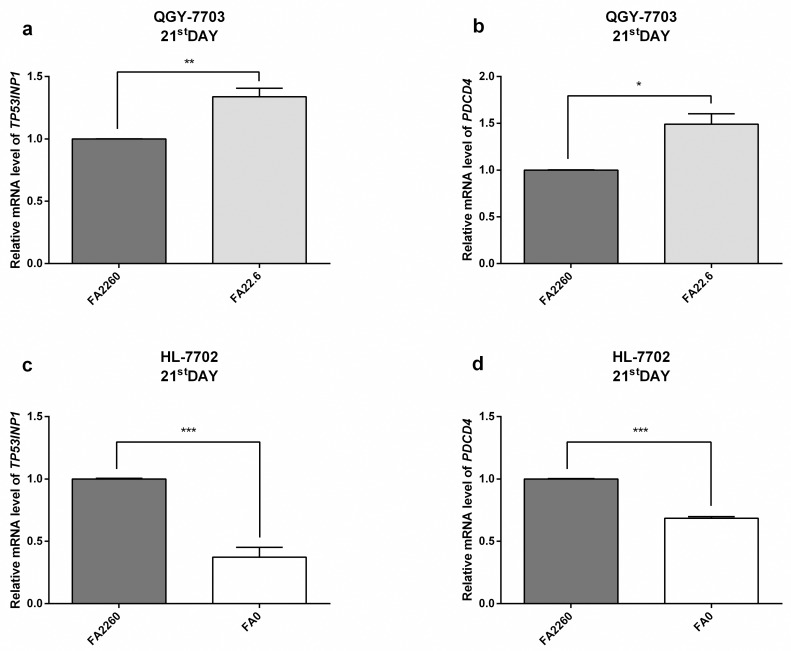
mRNA expression level of *TP53INP1*/*PDCD4* in QGY-7703/HL-7702 cells under conditions of FA deficiency/empty (21^st^ day); GAPDH served as an internal reference. (a) RT-qPCR assays reveal the mRNA expression of *TP53INP1* in QGY-7703 cells under conditions of FA deficiency on the 21^st^ day. (b) RT-qPCR assays reveal the mRNA expression of *PDCD4* in QGY-7703 cells under conditions of FA deficiency on the 21^st^ day. (c) RT-qPCR assays reveal the mRNA expression of *TP53INP1* in HL-7702 cells under conditions of FA empty on the 21^st^ day. (d) RT-qPCR assays reveal the mRNA expression of *PDCD4* in HL-7702 cells under conditions of FA empty on the 21^st^ day.

*TP53INP1* is a tumor suppressor gene induced with different stress conditions. *TP53INP1* overexpression leads to cell cycle arrest (G1 phase) and p53-dependent or independent apoptosis [36]. *PDCD4* is a tumor suppressor gene with negative regulation on the proliferation, metastasis, invasion and angiogenesis of Cancerous cells [37]. To further research the miR-22-3p and miR-149-5p induced biological meaning of the change in *MTHFR* expression in HCC cells, we added the expression of *TP53INP1* and *PDCD4* in HCC as two parameters under conditions of long term FA deficiency, and the results indicated that FA deficiency induced the expression of *TP53INP1* (34%) and *PDCD4* (49%) in elevated. On the contrary, the expression of *TP53INP1* and *PDCD4* in normal human hepatocytes (HL-7702) in decreased, and the results suggested that extreme FA deficiency might increase risk of human hepatocyte carcinogenesis. (Fig 5) The decline of *MTHFR* expression by miR-22-3p and miR-149-5p might inhibit methylation to positively regulate *TP53INP1* and *PDCD4*, thus promoting HCC cell apoptosis.

## Discussion

**Fig 6:**
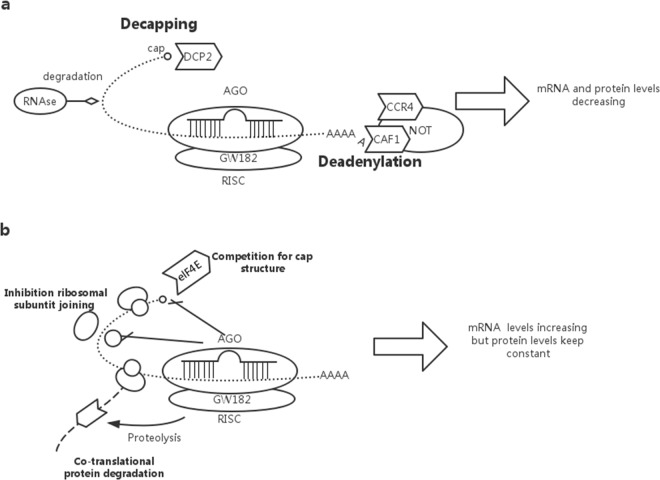
The mechanism might be different in regards to the miRNA-mediated mRNA silencing in HCC (a) and normal hepatocytes (b).

The results of this study suggested that miR-22-3p directly targeted *MTHFR*, which led to the repression of *MTHFR* expression. In addition, the Poly (A) Tailing RT-qPCR validation results showed that the expression of miR-22-3p and miR-149-5p was significantly increased in the normal human hepatocyte cell line HL-7702 and the hepatocellular carcinoma cell line QGY-7703 under conditions of FA deficiency compared with the control. Furthermore, a high-level expression of miR-22-3p and miR-149-5p in HCC and human hepatocytes was strongly associated with the downregulation of *MTHFR* mRNA levels under conditions of FA deficiency. Moreover, the slightly high expression of miR-22-3p and miR-149-5p was correlated with the maintenance of the translational levels of *MTHFR* in normal hepatocytes. In addition, the overexpression of miR-22-3p and miR-149-5p was associated with the inhibition of *MTHFR* protein levels in HCC under conditions of FA deficiency/FA-free medium. Importantly, our investigation indicated the possible presence of different mechanisms of miRNA-mediated *MTHFR* gene silencing;[38] One mechanism involves the following microRNA-mediated mRNA decay in HCC under the condition of FA deficiency: miRNAs trigger deadenylation and subsequent decapping of the *MTHFR* mRNA target, which leads to the inhibition of mRNA circularization and the promotion of mRNA degradation. The proteins required for this process are shown and include components of the major deadenylase complex (CAF1, CCR4, and the NOT complex), the decapping enzyme DCP2, and several decapping activators. The destruction of a mature mRNA means that the cytoplasmic poly (A) binding protein and the cytoplasmic cap-binding protein cannot be combined with mRNA, and thus the mRNA stability is reduced by RNase degradation. According to our result, the decreased mRNA levels of *MTHFR* detected in HCC might be mediated through this mechanism. Another mechanism involves the miRNA-mediated inhibition of translation in normal hepatocytes under FA-free conditions; this includes the inhibition of translation, the degradation of co-translation of proteins, the competition for the cap structure and the inhibition of ribosomal subunit assembly. In our study, this mechanism induced low protein levels of *MTHFR* and maintained a balance in the translation levels of *MTHFR*, as high mRNA levels of *MTHFR* were detected in normal hepatocytes. (Fig 6) We revealed that miR-22-3p and miR-149-5p potentially acted as important tumor suppressors by one-carbon metabolism in HCC.

**Fig 7:**
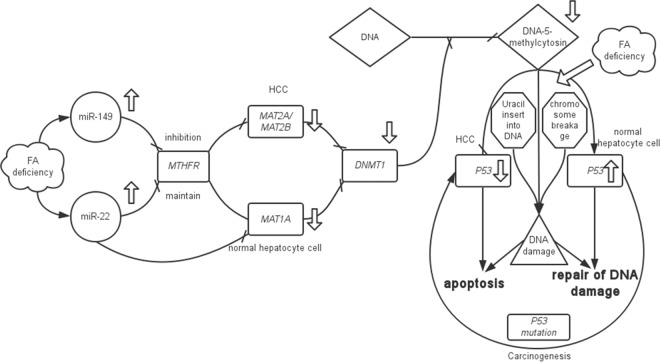
A schematic model illustrates how the upregulation of miR-22 and miR-149 might promote apoptosis (or not) in HCC cells (or normal hepatocytes) via the downregulation (or maintenance) of *MTHFR*, which causes *MAT1A* (or *MAT2A*/B) inhibition and the induction of genome hypomethylation. In contrast, FA deficiency promotes uracil misincorporation into human DNA and chromosome breakage; this causes DNA damage, which is repaired (or not) by *P53*.

To further decipher why FA deficiency induced the upregulation of miR-149 and miR-22 expression and inhibited the expression of *MTHFR* in HCC but maintained its expression in normal hepatocytes. In HCC, the reduced expression of *MAT1A* caused an increase in *MAT2A* expression[39] (*MAT2B* is coding a subunit of *MAT2A*). The inhibition of *MTHFR* downregulated *MAT2A*/*MAT2B* and decreased the expression of DNA (cytosine-5)-methyltransferase 1 (*DNMT1*). *DNMT1* is an enzyme that catalyzes the transfer of a methyl group to DNA, and thus, it affects whole-genome methylation. Genome hypomethylation associated with folate deficiency causes the misincorporation of uracil into human DNA and subsequent chromosome breakage [40], which leads to DNA damage. On the contrary, in tumor tissue, FA deficiency results in a decrease in *P53* transcription and is associated with relative hypermethylation[21]. If *P53* is decreased, DNA damage cannot be repaired, which induces tumor cell apoptosis. In normal hepatocytes, miR-22 and the inhibition of *MAT1A* expression [41] lead to an overall decrease in methylation. However, an increased level of *P53* mRNA is associated with hypomethylation in the coding region of the gene[21]. When *P53* is upregulated, DNA damage is restored by *P53* [42]. Once *P53* is mutated, DNA damage is not able to be repaired, which leads to carcinogenesis. With the above findings, our study provides a novel and comprehensive insight into the functional roles of miR-22-3p and miR-149-5p as they relate to the regulation of *MTHFR* in the development of HCC by DNA methylation and DNA synthesis/mismatch repair. (Fig 7) Altogether, these results provide additional insights that the dysregulation and instability of DNA methylation are mediated by miRNAs that accompany the transition of normal cells to tumor cells. This might be a reason why HCC cells cannot survive without FA.

Previous studies have suggested that miR-22 functions in multiple cellular processes, including proliferation, differentiation, senescence and apoptosis and that their dysregulation is a hallmark of human cancer [43]. MiR-22 has been determined to be a regulator or an inhibitor in diverse cancers, including osteosarcoma, prostate cancer, cervical cancer, lung cancer, [44]breast cancer[45], colorectal cancer[46], gastric cancer [47, 48], ovarian cancer[49], acute myeloid leukemia[50], medulloblastomas[51], endometrial endometrioid carcinomas[52], esophageal squamous cell carcinoma[53] and hepatocellular carcinoma[54]. The study by Zhou et al. [55] found that miR-22 is downregulated in HCC and that its expression is associated with the differentiation, metastasis and prognosis of carcinomas. They also found that the expression of miR-22 in hepatocellular carcinoma is significantly associated with histological differentiation and is negatively correlated with the expression of ezrin protein. *Histone deacetylase 4* (*HDAC4*) [56] and *Cyclin-dependent kinase inhibitor 1A* (*CDKN1A*) [57] are two critical proteins in cancer, and both are directly targeted and regulated by miR-22. The level of miR-22, which is downregulated in HCC, is correlated with a decrease in the disease-free survival of HCC patients. The survival time is shorter in patients with low miR-22 expression compared with those with high expression, and the overexpression of miR-22 exerts an anti-proliferative effect on HCC cells both in vitro and in vivo. Zekri AN et al.[58]identified serum miR-22 and other miRNAs with alpha fetoprotein (AFP) that might be of considerable clinical use in the early detection of HCC in both normal populations and high-risk patients. They might be of use by providing high diagnostic accuracy (the area under the receiver operating characteristic curve (AUC) = 0.982) for the early detection of HCC in liver cirrhosis (LC) patients, high diagnostic accuracy (AUC = 0.988) for the early detection of HCC in chronic hepatitis C (CHC) patients and high diagnostic accuracy (AUC = 1) for the discrimination between the LC group and the control group.

MiR-149 is a potential prognostic marker for gastric cancer [59], upper tract urothelial carcinoma [60], colorectal cancer [61] and hepatocellular carcinoma. Clinical evidence also highlights the prognostic potential of miR-149 in HCC [62]. Pre-miR-149 rs71428439 may be a genetic risk factor for HCC, as miR-149 expression in patients with the GG genotype was shown to be significantly lower compared with patients with the AG or AA genotypes. Additionally, tumor tissues from patients with the GG genotype show increased *AKT1* and *Cyclin D1* expression compared with tumor tissues from patients with the AA and AG genotypes. [63] A meta-analysis showed that the miR-149 gene rs2292832 polymorphism contributes to the risk of hepatocellular carcinoma [64]. The overexpression of miR-149 or the inhibition of *PARP-2* expression can inhibit tumor growth, which is more effective in the sensitization of animals with HCC xenografts to chemotherapy and radiotherapy[65]. MiR-149 suppresses HCC cell invasion and metastasis via the suppression of *Mg2+/Mn2+-dependent*, *1F* (*PPM1F*), which mediates the formation of stress fibers in vivo [66]. Wu J et al.[63]demonstrated that one mechanism possibly involves the downregulation of miR-149 expression and the upregulation of *AKT1* expression. The targeting of *AKTs* by miR-149 reveals the critical role of this miRNA in the malignant progression of HCC via the modulation of the *AKT*/*mTOR* pathway [62]. Lu X et al.[67]confirmed that Bafilomycin A1 inhibits the growth and metastatic potential of the BEL-7402 liver cancer cells and induces miR-923, miR-1246, miR-149, miR-638 and miR-210 upregulation, which may represent promising targets for anti-cancer therapies.

*MTHFR* is a key gene of OCM and is a key enzyme involved in DNA methylation and DNA synthesis/mismatch repair. The deletion of *MTHFR* decreases the transfer of methyl groups by *DNMT1*, which complicates the process of DNA methylation. Maivel Ghattas et al.[68] suggested the possible involvement of *MTHFR* promoter methylation and the consequent inactivation of *MTHFR*, which might be part of a feedback regulation mechanism of *MTHFR*. Decreased *MTHFR* level induced DNA hypomethylation and Ding, X. et al.[69] indicated that the methylation rate of *PDCD4* promoter is significantly higher in HCC tissues than that in adjacent nontumor tissues. *PDCD4* mRNA levels and promoter methylation levels are both statistically correlated with metastasis and the degree of differentiation in HCC. Weng W et al.[70] found that hypomethylation of *TP53INP1* promoter region correlate with silencing of *TP53INP1* contribute to the pathogenesis of human cancer. Mikael LG et al.[71]considered that disturbances in FA metabolism or methylation reactions may also play a critical role. Tannapfel A[72] demonstrated that DNA methylation is a possible biomarker for the early detection of HCC. The reversibility of methylation also suggests that this mechanism may be used as a treatment option in the future. Upon comparison of 1500 cases of HCC, 1500 healthy controls and individuals with the wild-type homozygous genotype, Zhang H et al.[73]indicated that genetic variants in OCM genes (including *MTHFR*) may contribute to HCC susceptibility, as those with the heterozygous genotype had a higher HCC risk. Qi YH et al.[74]reported that homozygous carriers of the *MTHFR* C677T mutation are more susceptible to HCC, but homozygous mutations of *MTHFR* A1298C may play a protective role in the development of HCC. More interestingly, the protective role of the *MTHFR* A1298C polymorphism is more obvious when the controls examined are healthy individuals. Most importantly, the study by Yu MC et al.[75] indicated that low-activity genotypes (reduced enzymatic activities) of *MTHFR* and high-activity genotypes (enhanced enzymatic activities) of *thymidylate synthase* (*TYMS*), both of which discourage the misincorporation of uracil into DNA, are associated with a reduced risk of HCC.

## Conclusion

In summary, our results first established *MTHFR* as a direct functional effector of miR-22-3p and miR-149-5p in HCC. We found that miR-22-3p and miR-149-5p are potent tumor suppressors in HCC. The upregulation of miR-22-3p and miR-149-5p promotes the dysregulation of DNA methylation and DNA synthesis/mismatch repair in HCC via the downregulation of *MTHFR* in conditions of FA deficiency. The determination of two types of potential mechanisms of miRNA-Mediated *MTHFR* gene silencing in the normal human hepatocyte cell line HL-7702 and the human hepatocellular carcinoma cell line QGY-7703. FA deficiency involves the imbalance of DNA methylation status, which induces the inactivation of *MTHFR* and *P53* and other genetic damage. To the best of our knowledge, this is the first study to demonstrate that the miR-22-3p/miR-149-5p/*MTHFR* axis potentially regulates the DNA methylation/synthesis/mismatch repair system of HCC cells and normal hepatocytes (HL-7702 cells). These findings also provide a better understanding of the development and progression of HCC, and might be an important implication for future therapy in patients with HCC.

However, further studies are required to determine whether the examined miRNAs exhibit similar effects in other types of solid tumors.
